# Hemodynamic predictors of aortic dilatation in bicuspid aortic valve by velocity-encoded cardiovascular magnetic resonance

**DOI:** 10.1186/1532-429X-12-4

**Published:** 2010-01-13

**Authors:** P Martijn den Reijer, Denver Sallee, Petra van der Velden, Eline R Zaaijer, W James Parks, Senthil Ramamurthy, Trevor Q Robbie, Giorgina Donati, Carey Lamphier, Rudolf P Beekman, Marijn E Brummer

**Affiliations:** 1 Leiden University Medical Center, Leiden, The Netherlands; 2 Sibley Heart Center Cardiology, Atlanta, Georgia, USA; 3 Children's Healthcare of Atlanta, Atlanta, Georgia, USA; 4 Emory University School of Medicine, Atlanta, Georgia, USA

## Abstract

**Background:**

Congenital Bicuspid Aortic Valve (BAV) is a significant risk factor for serious complications including valve dysfunction, aortic dilatation, dissection, and sudden death. Clinical tools for identification and monitoring of BAV patients at high risk for development of aortic dilatation, an early complication, are not available.

**Methods:**

This paper reports an investigation in 18 pediatric BAV patients and 10 normal controls of links between abnormal blood flow patterns in the ascending aorta and aortic dilatation using velocity-encoded cardiovascular magnetic resonance. Blood flow patterns were quantitatively expressed in the angle between systolic left ventricular outflow and the aortic root channel axis, and also correlated with known biochemical markers of vessel wall disease.

**Results:**

The data confirm larger ascending aortas in BAV patients than in controls, and show more angled LV outflow in BAV (17.54 ± 0.87 degrees) than controls (10.01 ± 1.29) (p = 0.01). Significant correlation of systolic LV outflow jet angles with dilatation was found at different levels of the aorta in BAV patients STJ: r = 0.386 (N = 18, p = 0.048), AAO: r = 0.536 (N = 18, p = 0.022), and stronger correlation was found with patients and controls combined into one population: SOV: r = 0.405 (N = 28, p = 0.033), STJ: r = 0.562 (N = 28, p = 0.002), and AAO r = 0.645 (N = 28, p < 0.001). Dilatation and the flow jet angle were also found to correlate with plasma levels of matrix metallo-proteinase 2.

**Conclusions:**

The results of this study provide new insights into the pathophysiological processes underlying aortic dilatation in BAV patients. These results show a possible path towards the development of clinical risk stratification protocols in order to reduce morbidity and mortality for this common congenital heart defect.

## Introduction

Bicuspid aortic valve (BAV) is the most common congenital cardiovascular malformation with an estimated prevalence of 1-2% [1,2] in the general population, and is a significant risk factor for several serious complications including aortic dilatation and dissection [1,3,4]. An association between aortic dilatation and BAV has been confirmed in numerous studies [5-14] in comparisons against normal controls. Aortic dilatation, initially asymptomatic in BAV, often develops during childhood [8,12,15]. Dilatation may be observed at different levels in the aorta, but most commonly involves the ascending aorta [14,16]. BAV patients with a dilated aorta have a 9-fold increased risk of aortic dissection [3,17] and have an increased risk of sudden cardiac death, even after valve replacement [18-20]. However, the progression of aortic dilatation varies considerably between BAV patients, ranging from normal size development to severely dilated at early ages [12]. This complicates accurate prognosis at the time of identification of the lesion and cost-effective tailoring of follow-up recommendations.

Even though the association between bicuspid aortic valves and aortic dilatation is well known, clinical predictors for the development and progression of aortic dilatation are lacking. Abnormal blood flow patterns and the consideration of BAV as part of a connective tissue disease may be important factors, but their relative importance remains unclear. Excised BAVs have been shown associated with abnormal flow patterns and turbulence aimed toward the right anterolateral wall (convexity) of the aorta in flow loop experiments with phantom models of aorta [21]. An echocardiographic study involving aortic tissue Doppler has demonstrated greater hemodynamic stress on the anterolateral ascending aorta [22]. Recently, the presence of abnormal aortic blood flow patterns was confirmed *in vivo *by 4-D cardiovascular magnetic resonance (CMR) visualization in case reports involving BAV in association with a coarctation [23] and in a case of BAV with ascending aortic aneurysm [24]. Pathological changes in smooth muscle cells and associated extracellular matrix protein expression have been found to be most significant in the convexity of the aorta, where wall stress due to abnormal flow patterns is expectedly highest [25,26]. Local variations have been found in thickness and mechanical properties of excised ascending aortas [27]. In BAV patients the outer curvature had reduced elasticity and AA wall thickness compared to subjects with tricuspid aortic valves. Accordingly, both abnormal aortic blood flow patterns and abnormal extracellular matrix protein expression could have value as potential predictive tools for clinical risk stratification.

This paper reports on a prospective CMR study to define quantifiers of abnormal aortic blood flow patterns in a group of pediatric BAV patients, in comparison to normal control subjects in the same age range. The main purpose of the study is to investigate the hypothesis that more eccentric systolic aortic flow jets in BAV patients are associated with more dilated aortic dimensions. The study was motivated by the need for a robust risk stratification protocol for development of clinically significant complications in pediatric BAV patients. Support for the stated hypothesis could help justify more extensive investigation of blood flow phenomena in this context. The same motivation also prompted us to add to the experimental protocol a confirmatory investigation of serum levels of Matrix Metalloproteinases (specifically MMP2 and MMP9) and associated tissue inhibitors (specifically TIMP1 and TIMP2). MMPs are associated with various forms of vascular remodeling [28-32], and are readily quantified by commercially available assays. Serum levels of MMP and TIMP have been shown altered in BAV [33], especially with severe aortic dilatation [34], and thus may be investigated as potential low-cost, non-specific biomarkers of vessel wall injury in BAV.

## Methods

Time-resolved structural and 3-D velocity-encoded CMR images were visualized jointly to create a dynamic 3-D view of blood flow patterns as vector fields in an anatomical context using CMR techniques similar to those explored previously for observation of blood flow patterns in the great arteries [35-40], brain [41] and abdomen [23,42]. First we calculated the expected ideal systolic flow jet direction vector, measured as the aortic channel direction in structural CMR images of the left ventricular outflow tract (LVOT). Subsequently the actual direction vector of the flow jet was determined from the flow velocity data as the average systolic peak forward flow direction. The resultant angle between these two direction vectors represents a quantitative measure of misdirected blood flow. This angle was correlated with aortic dilatation at four commonly used anatomical levels: the aortic valve plane (AOV), sinus of Valsalva (SOV), sinotubular junction (STJ), and the ascending aorta (AAO). In addition, for the BAV patients in our study, aortic dilatation and misaligned blood flow were also correlated with plasma levels of the extracellular matrix proteins MMP-2, MMP-9, TIMP-1 and TIMP-2, which have been previously proposed by other investigators as non-imaging markers of vessel wall disease [29-32].

### Study Population

From October 2006 until July 2008 a total of 18 asymptomatic BAV patients and 11 healthy control subjects in the age group 10-18 years were recruited and successfully underwent CMR. All subjects gave informed consent/assent per IRB-approved protocol. Patient exclusion criteria were: systemic hypertension for age and height, treatment with cardiac or vasoactive medications, subjects suffering from possibly confounding conditions such as connective tissue diseases including Marfan or Turner syndrome, greater than mild aortic stenosis (defined by a mean gradient by Doppler echocardiography of ≥ 17 mmHg), more than mild aortic regurgitation (defined by a regurgitant jet resulting in a left ventricular end-diastolic dimension >2 standard deviations above the mean for body surface area (BSA), or the presence of pan-diastolic retrograde flow on pulsed Doppler evaluation of the abdominal descending aorta). One control subject was found to have dilatation of the ascending aorta, considered unrepresentative of the normal pediatric population, and was excluded from analyses. General patient characteristics are summarized in Table 1.

**Table 1:** Baseline characteristics of BAV patients and control subjects.

Characteristic	BAV patients	Controls	p-value
N	18	10	
Age	15.7 +/- 1.9	14.2 +/- 2.6	0.10
BSA	1.7 +/- 0.30	1.7 +/- 0.31	0.87
male	72.2 % (n = 13)	70 % (n = 7)	0.87
female	27.8 % (n = 5)	30 % (n = 3)	
			
**Characteristic**	**BAV Patients**		
		
Aortic dilatation	14		
Aortic aneurysm	0		
Left/right cor. cusp fusion:	15		
Right/non-cor. cusp fusion	3		
Left/non-cor. cusp fusion	0		

Standard deviations in the observed values are listed with subject age and BSA. P-values show no significant differences in estimated mean values between patients and controls.

### CMR

**Figure 1:**
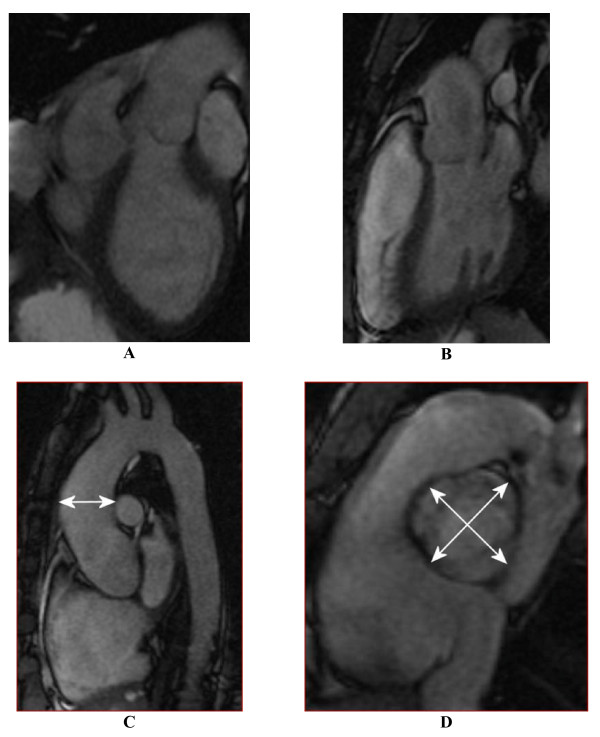
**Structural CMR images used in image analysis: (A) an "LVOT1" orthogonal view centrally in the LVOT, (B) a second orthogonal "LVOT2" view, (C) a "candy-cane" view of aortic arch, and (D) a systolic mid-SOV level view of valve leaflets**. Figures (A,B) are used to calculate the aortic channel direction, (C) illustrates AAO size measurement, and (D) illustrates how aortic sizes are measured at the levels of AOV, SOV, and STJ.

All subjects underwent a single non-contrast CMR which included structural and phase velocity-encoded imaging of the left ventricular outflow tract (LVOT) and ascending aorta. Structural breath-held SSFP ciné images were acquired on a GE Signa Twinspeed 1.5 T scanner, running Release 12M4. Scan parameters for the FastCINE/FIESTA pulse sequence included TR = 4 ms, TE = 2 ms, flip angle 45 degrees, typical FOV 300-350 mm, 128-192 phase encodings, RFOV 70-100%, reconstruction matrix 256 × 256, pixel BW 1 kHz, VPS = 8-12 (eff. temporal resolution 32-48 ms), 20 cardiac phases reconstructed using retrospective VCG gating, 1 slice acquired per breath-hold, ASSET parallel imaging acceleration used as SNR allowed, acquisition time 6-14 s/slice. Views included an oblique sagittal LVOT view (Figure 1A), an oblique coronal LVOT view (Figure 1B), a candy-cane view centrally through the aortic arch (Figure 1C), and views perpendicular to the aorta at the levels of AOV plane (Figure 1D), mid-SOV, STJ, and a more distal view through the mid-AAO. Time-resolved flow velocity encoded imaging was performed in breath-hold mode, using a FastCINE PC protocol with typical parameters TR = 12 ms, TE = 4 ms, flip angle 20 degrees, FOV 350 mm, RFOV 70%, 96-128 phase encodings, reconstruction matrix 256 × 256, pixel BW 125 Hz, VPS = 4-6, velocity encoding with VENC = 200 cm/s in all three dimensions (eff. Temporal resolution 192-288 ms), reconstructed at 20 time points in the cardiac cycle. Maxwell correction for concomitant gradient effects and gradient warp correction were performed by the GE scanner reconstruction software. Seven to ten equidistant parallel 2-D slice planes were scanned perpendicular to the valve plane, each acquired within a breath-hold interval with 3-D phase velocity encoding. The 7-9 mm thick slices, spaced 8-10 mm, cover the ascending aorta from just beneath the aortic valve plane into the transverse arch. 4-D flow imaging data allow better spatial and temporal resolution than FastCINE PC slice flow data. Therefore a 4-D flow scan[38,43] was also acquired on all subjects. However due to ethical concerns no gadolinium contrast was used in this study, and without contrast these scans suffer from poor contrast and SNR. For this reason these data were not used in this study.

### Image Analysis and Visualization

**Figure 2:**
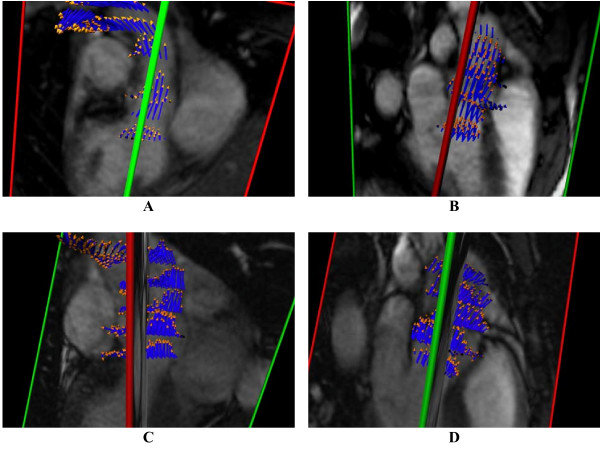
**3-D reconstructions of aortic blood flow during peak systole (A,B) in a normal subject with tricuspid aortic valve, showing a centrally directed systolic flow jet, and (C,D) in a BAV patient with aortic dilatation, showing anteriorly directed off-axis systolic flow**. The images in each pair of views mutually cross-reference the location of the perpendicular slice by the color of the intersection lines. The intersection line is ideally located along the LVOT channel axis but this scan planning does not necessarily work out perfectly in all cases, so in the analyses this direction is recalculated (see Figure 3).

Dynamic structural images and blood flow velocity patterns were visualized using the in-house developed Heartviz/Flowviz 3-D software. Example visualizations are shown in Figure 2. Aortic regions of interest (ROI) were identified in structural images and visualized jointly with the corresponding blood flow velocities, reconstructed from velocity-encoded images, and displayed as dynamic vector arrow fields.

#### Aortic Blood Flow Direction

**Figure 3:**
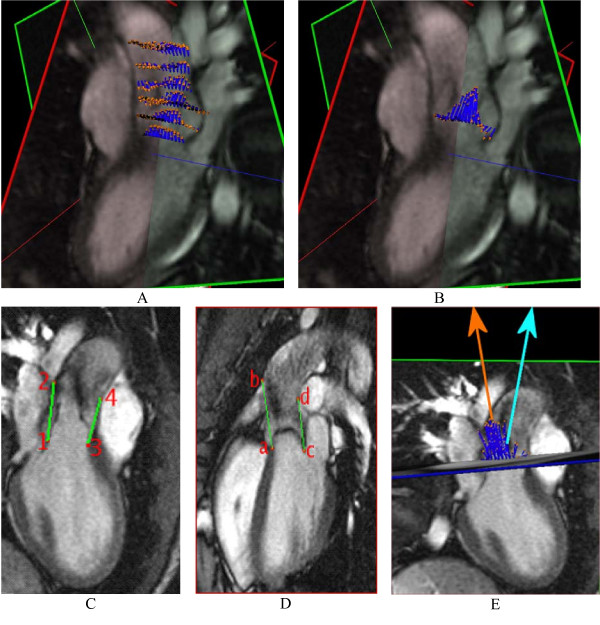
**Image analysis for calculation of the systolic flow jet angle: (A) 3-D view showing systolic flow velocity vectors in all slices**. (B) Systolic flow jet velocities in the central-most slice in the SOV, which are averaged to determine the flow jet direction (orange in E); (C,D) show the aortic channel direction definitions in the LVOT views. Line segments *1-2 *and *3-4 *in the LVOT1 view (A) and *a-b *and *c-d *in the LVOT2 view (B) are manually drawn to connect the proximal and distal boundaries on both sides of the SOV, which are easily detected landmarks. The vector average of each segment pair defines the projection of the outflow channel direction onto each image plane. These two projections are then used to calculate the channel axis (light blue in E) for calculation of the angle between the flow jet direction vector and channel axis, which quantifies the mis-direction of systolic flow.

Time points of systole and diastole were determined by observation of dynamics of cardiac contraction and blood flow. A vector representing direction and velocity of the peak systolic blood flow jet was calculated by averaging of flow vectors in all forward flow pixels inside the aorta (defined by manual ROI tracing in the associated image) in a slice located centrally in the sinuses of Valsalva at peak systole. Figure 3A shows systolic pixel flow vectors in six slices for a BAV patient, and Figure 3B shows the mid-SOV slice used for calculation of the flow jet direction, with the flow vectors amplified compared to (3A). A second vector representing the axis of the LVOT at systole was calculated from manually defined landmarks indicating the proximal valve plane and the sino-tubular ridge in the most central slices of the two orthogonal views of the LVOT (Figure 3C and 3D). The angle between the blood flow jet vector and the outflow tract axis, proposed as a quantitative parameter of mis-directed valvular flow, was then calculated from these vectors (Figure 3E).

#### Aortic diameter measurements

Aortic diameters were measured at the AOV, SOV, STJ and AAO during peak systole. At each location the largest observed perpendicular cross-sectional diameter was used for analysis. For diameters at the level of AOV, SOV, and STJ axial images at t hose locations were analyzed, angled perpendicular to the aorta (Figure 1D). The maximum diameter in the AAO was determined as the largest cross-section observed perpendicular to the aortic axis curve in a mid-vessel slice in the "candy-cane" view (Figure 1C), distal to the STJ and proximal to the branch of the brachiocephalic trunk. For statistical analysis all aortic diameters were corrected for Body Surface Area (BSA) by dividing diameters by the square root of the BSA (Haycock formula [44]).

### MMP 2, 9 and TIMP 1, 2 plasma measurements

From the BAV patients blood samples were obtained prior to CMR from peripheral veins and collected in EDTA tubes. These measurements were not obtained in control subjects in this preliminary study for reasons of ethics and recruitment logistics. Plasma was separated by centrifugation at 3300 rpm for 10 min at 4°C and immediately frozen and stored at -80°C. Plasma MMP2, MMP9, TIMP1 and TIMP2 levels were determined by two-site enzyme-linked immunosorbent (ELISA) assays using commercially available kits with monoclonal antibodies against the proteins (Amersham Human Biotrak; GE healthcare Bio-Sciences, Piscataway, NJ). The assays measured total MMP 2, MMP 9 and TIMP1 and TIMP 2 levels in the samples. All samples were run in duplicate and results were averaged.

### Statistics

Distribution characteristics are presented as mean +/- standard error of the mean, unless otherwise noted. Distributions were tested for normality using Q-Q plots and the Shapiro-Wilk test. Group comparisons between BAV patients and controls used the unpaired Student's t-test. Pearson's correlation coefficients were used to determine correlations between normal distributions. Spearman's non-parametric rank correlation coefficients were used for very small samples and non-normally distributed populations.

Prior to all correlation analyses data were screened on multivariate outliers using informal identification in scatter plots and calculation of Mahalanobis distance. Outliers were not removed from data. All tables indicate the number of subjects on which results are based.

Intra-observer variability in aortic dimension and flow angle measurements was calculated by evaluation of duplicate measurements by the same observer. Inter-observer variability was calculated by evaluation of the same measurements by two observers. These measurement sets were evaluated for the entire study population, and thus represent the entire encountered range of sizes and blood flow. Calculated statistics included Lin's concordance correlation coefficients (CCC) [45], the estimated standard deviation, and where appropriate, the associated coefficient of variation (CV), and Bland Altman 95% confidence limits of agreement [46].

Statistics were evaluated using Microsoft Excel 2003 (Microsoft, Redmond, WA) and SPSS 16.0 for Windows (SPSS Inc., Chicago, Illinois). A p-value < 0.05 was considered to establish statistical significance.

## Results

Characteristics of all BAV patients and control subjects are summarized in Table 1. The gender distribution in the study was consistent with the approximately 2:1 male predominance of BAV found in literature [9]. No significant group differences were found in BSA or age between patients and controls. Fifteen of the 18 BAV patients had a fusion of the left and right coronary cusps, 3 had a fusion of the right and non-coronary cusps, and none had a fusion of the left and non-coronary cusps; 14 patients had a diagnosed dilated ascending aorta (defined as >2 standard deviations above normal size in echo, BSA-adjusted); None had a diagnosed aortic aneurysm (commonly defined subjectively as having BSA-adjusted size 50% above normal [47]), one had stable mild aortic regurgitation, one had a small apical muscular ventricular-septal defect, and one had suspected mild sinus node dysfunction. None of the complications warranted exclusion of the subject.

### Aortic diameters

**Figure 4:**
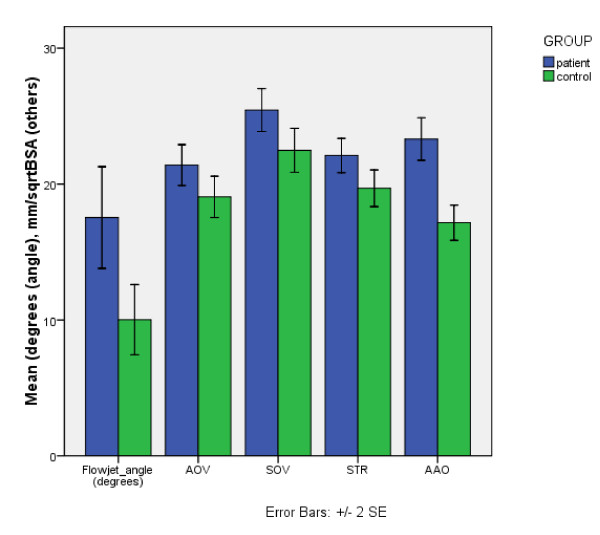
**Comparison of mean flow jet angle and aortic diameters, adjusted for BSA, between BAV patients and control subjects. Errors bars indicate two standard errors of the mean**.

Comparison of aortic diameters, corrected for BSA, revealed that on average BAV patients had a statistically significant larger diameter at the level of the SOV, STJ and AAO compared to controls (Figure 4, Table 2). The difference in diameter at the level of the AOV approached statistical significance. We found that the aortic diameters were normally distributed at all four levels.

**Table 2:** Mean diameters at all 4 aortic levels in BAV patients and control subjects, adjusted for BSA.

	BAV			Controls			
	
	N	Mean	Mean/√BSA	N	Mean	Mean/√BSA	p-value
AOV (mm)	18	27.95 +/- 1.06	21.40 +/- 0.75	10	24.71 +/- 1.15	19.05 +/- 0.76	0.054
SOV (mm)	18	33.17 +/- 1.09	25.44 +/- 0.79	10	29.01 +/- 0.77	22.48 +/- 0.81	0.022
STJ (mm)	18	28.90 +/- 1.00	22.09 +/- 0.63	10	25.59 +/- 1.25	19.69 +/- 0.67	0.022
AA (mm)	18	30.79 +/- 1.18	23.31 +/- 0.78	10	22.96 +/- 1.26	17.15 +/- 0.65	<0.001

ANGLE (°)	18	17.54 +/- 0.87		10	10.01 +/- 1.29		0.01

Given are standard errors of the mean. Significance p-values reflect differences in mean BSA-adjusted diameter between BAV patients and control subjects.

**Figure 5:**
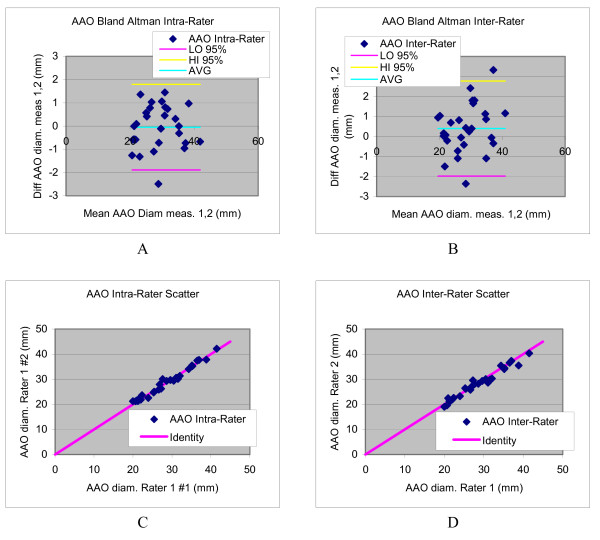
**Bland Altman plots (a,b) and scatter diagrams (c,d) illustrating the intra- and inter-rater variability in ascending aorta size evaluations**.

The inter- and intra-observer variability for diameter measurements is summarized in Table 3. Reproducibility of the aortic size parameters was generally excellent, with CCC values all above 0.89, and coefficients of variation ranging between 2.3 and 5.1%. Bland Altman plots and scatter diagrams for reproducibility of the ascending aorta are shown in Figure 5. Bland Altman analysis shows in all cases confidence intervals that include zero, indicating absence of evidence of observer bias.

**Table 3:** Reproducibility statistics for intra-rater and inter-rater variations in measured aortic diameters and flow angles.

		Intra-Rater			
	**Mean Value (mm)**	**Mean Stddev (mm)**	**CV (%)**	**CCC**	**Bland Altman 95% confidence interval of difference (mm)**

**AOV**	23.74	1.17	4.6%	0.926	[-2.59,3.91]

**SOV**	30.32	0.98	3.2%	0.949	[-2.44,3.00]

**STJ**	27.42	0.67	2.6%	0.970	[-2.14,1.59]

**AA**	27.91	0.67	2.3%	0.988	[-1.89,1.79]

	**Mean Value (degrees)**	**Mean Stddev (degrees)**	**CV (%)**	**CCC**	**Bland Altman 95% confidence interval of difference (degrees)**

**FLOW ANGLE**	13.7	1.64	12.0%	0.946	[-4.34,4.77]

		**Inter-Rater**			

	**Mean Value (mm)**	**Mean Stddev (mm)**	**CV (%)**	**CCC**	**Bland Altman 95% confidence interval of difference (mm)**

**AOV**	23.74	1.33	5.1%	0.893	[-4.94,2.45]

**SOV**	30.32	1.23	3.9%	0.914	[-4.17,2.62]

**STJ**	27.42	1.00	3.8%	0.910	[-3.76,1.79]

**AA**	27.91	0.86	3.0%	0.977	[-1.97,2.78]

	**Mean Value (degrees)**	**Mean Stddev (degrees)**	**CV (%)**	**CCC**	**Bland Altman 95% confidence interval of difference (degrees)**

**FLOW ANGLE**	13.7	1.38	10.2%	0.960	[-3.40,4.23]

Data are based on 2 repeat measurements in all (25) subjects. The table gives the estimated standard deviation in repeat measurements based on pair-wise differences, the mean corresponding coefficient of variation, the concordance correlation coefficient{Lin, 1989 #188}, and Bland Altman confidence intervals{Bland, 1986 #246}.

### Direction of Systolic Aortic Flow Jet

The average angle between the systolic blood flow jet direction at the level of the AOV and the left ventricular outflow tract axis was significantly larger in BAV patients as a group (17.54 +/- 0.87 degrees) than in control subjects (10.01 +/- 1.29 degrees, p = 0.01) (Table 2). The distributions of angle measurements passed the test of normality.

The blood flow jet angle and aortic diameter showed significant r-values in BAV patients (Table 4) at the level of the STJ (p = 0.048) and the AAO (p = 0.022) but r-values were not significant in correlations with aortic dimensions at any level in control subjects (Table 5).

**Table 4:** Pearson correlations between blood flow jet angle and aortic diameter at the indicated aortic levels in BAV patients only.

	N	r-value	p-value
AOV	18	0.000	0.999
SOV	18	0.386	0.114
STJ	18	0.472	0.048*
AA	18	0.536	0.022*

An asterisk marks statistical significance at the 95% probability level.

**Table 5:** Pearson correlations between blood flow jet angle and aortic diameter at the indicated aortic levels in control subjects only.

	N	r-value	p-value
AOV	10	-0.129	0.999
SOV	10	-0.378	0.282
STJ	10	0.377	0.283
AA	10	0.243	0.498

**Figure 6:**
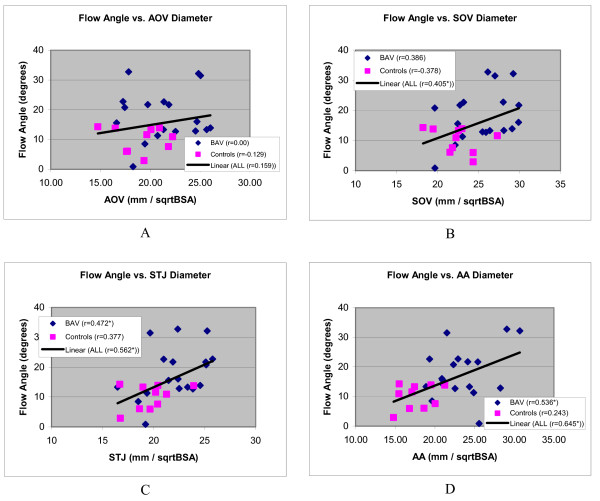
**Scatter diagrams showing distribution systolic blood flow jet angles of aortic outflow as a function of aortic diameters, corrected for BSA, and at the level of the aortic valve (A), sinus of Valsalva (B), sinotubular junction (C) and ascending aorta (D)**. Regression lines are shown of line fits with all subjects combined. An asterisk with the Pearson correlation coefficient in the figure legend indicates a statistically significant correlation.

If eccentric systolic blood flow causes dilatation, such an effect may manifest regardless of the immediate cause of such flow patterns, and may thus not be limited to BAV subjects. When BAV patients and control subjects were combined into one group (Table 6), the blood flow jet angle was indeed more strongly correlated to aortic diameters. In the pooled populations we found significant r-values at the levels of the SOV (p = 0.023), STJ (p = 0.014) and AAO (p = 0.001). Figure 6 further shows scatter diagrams of the distributions for BAV patients and controls at all four levels.

**Table 6:** Pearson correlations between blood flow jet angle and aortic diameter at the indicated aortic levels.

	N	r-value	p-value
AOV	28	0.159	0.420
SOV	28	0.405	0.033*
STJ	28	0.562	0.002**
AA	28	0.645	<0.001**

BAV patients and control subjects were combined in one group for these results. An asterisk marks statistical significance at the 95% probability level; two asterisks mark significance at the 99% level.

**Figure 7:**
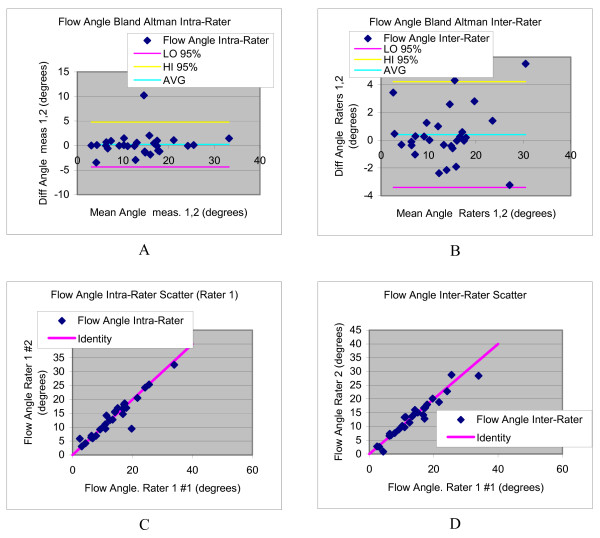
**Bland Altman plots (a,b) and scatter diagrams (c,d) illustrating the intra- and inter-rater variability in flow angle evaluations**.

The inter- and intra-observer variability for flow jet angle measurements is summarized in Table 3. Intra- and inter-rater reproducibility of the flow jet angle was good, with CCC values of 0.946 and 0.960, respectively. The average flow angle was 13.7 degrees, and estimated standard deviations in repeat measurements of 1.64 degrees (intra-rater) and 1.38 degrees (inter-rater), resulting in CV values of 12.0% and 10.2%, respectively. The Bland Altman analysis is illustrated in Figure 7.

### Plasma protein levels

Plasma samples were collected from all 18 BAV patients and MMP2, MMP9, TIMP1 and TIMP2 plasma levels were measured using an ELISA assay. The mean MMP2 concentration was 1387.5 +/- 82.4 ng/ml, mean MMP9 concentration was 8.85 +/- 1.6 ng/ml, TIMP1 concentration was 268.9 +/- 13.8 ng/ml and mean TIMP2 concentration was 80.3 +/- 7.1 ng/ml.

Except for TIMP1 none of the distributions of concentration measurements passed the test for normality. For this reason correlations between protein levels and aortic diameters were determined using non-parametric Spearman's correlation coefficients. MMP 2 plasma levels were significantly correlated to aortic diameter at the level of the AAO (r = 0.512, P = 0.025) and approached significant correlations at the levels of the SOV (Spearman rank r = 0.430, p = 0.066) and STJ (r = 0.444, p = 0.057). TIMP2 plasma levels correlated significantly to aortic diameter at the level of the STJ (r = 0.570, p = 0.011) and moderately at the level of the SOV (r = 0.458, p = 0.049). MMP9 and TIMP1 plasma concentrations were not correlated to aortic diameter at any anatomical level.

Plasma MMP2 levels were moderately correlated to blood flow jet angle (Spearman r = 0.509, p = 0.044). No significant correlation was observed between blood flow jet angle and MMP9 or TIMP1 and TIMP2 plasma levels.

## Discussion

We found significant correlations between the blood flow jet angle, representing a quantitative measure of misdirected blood flow, and aortic diameters at the level of the SOV, STJ and AAO. The significantly positive correlation coefficients suggest that the larger the angle of misdirected flow with the aortic axis, the larger aortic diameter and thus the more severe the observed dilatation is. These results favor the hypothesis that abnormal, misdirected blood flow patterns associated with BAV are directly implicated in the development of aortic dilatation and that dilatation is not solely a manifestation of a connective tissue disease as some investigators have proposed [48]. Correlations are most significant at the more distal level of the ascending aorta, where shear wall stress and pressure are expectedly highest.

In addition to the flow data, we also found significant correlations between aortic diameters and MMP 2 and TIMP 2 but not MMP 9 and TIMP 1 plasma levels in BAV patients. These results confirm previous studies that demonstrated significantly increased tissue levels of MMP 2 but not MMP 9 in aneurysms of BAV patients compared to TAV patients [34,49,50], and our results confirm an earlier study that reported a significant correlation between aortic diameter and MMP 2 expression [49]. In addition, by our best knowledge this study is the first to also demonstrate a relation between TIMP 2 plasma levels and aortic diameter. Although it remains inconclusive from our results whether elevated MMP 2 and TIMP 2 plasma levels are a cause or a result of aortic dilatation, the results indicate that MMP levels and fluid-mechanical forces due to abnormal flow patterns both play a role in the development of vessel wall disease. More extensive studies will be needed to determine the weight of these important causal contributors to aortic dilatation.

The hypothesis of a direct relation between abnormal flow patterns and aortic dilatation is consistent with our observations that the correlation between the blood flow jet angle and aortic diameter is stronger when BAV patients and control subjects are pooled into a single population. A hypothesized effect of dilatation as a result of eccentric systolic blood flow need not necessarily be specific to BAV subjects, but may occur similarly in other individuals with similarly disturbed flow, and absent in any subjects with well-aligned flow patterns. In this respect it is a logical step to consider both groups as different classes within one population, who are all subject to this same effect. With the relatively small number of subjects included in the present study significant correlations were found at 2 of 4 levels of the aorta when observing BAV patients alone. The normal population alone mostly has normal flow, hence lower spread in aortic sizes, and thus shows no correlation. Pooling the normal control subjects and the BAV population will show larger numbers as well as an increased range of both phenomena, evidenced by a stronger correlation, which is now significant at 3 out of 4 levels of the aorta. Again larger studies in a wider age range are required to actually determine cause and effect and to differentiate between BAV patients at risk for rapid aortic dilatation and lower-risk slow progressors. The causality question may of course not be straightforward to answer, since it is well-known that BAV is also associated with connective tissue disease [33,51,52]. Both these (and likely also other) factors may in actuality play a causal role.

Should a causal link between misdirected blood flow and severity of aortic dilatation be confirmed, a combination of imaging tests and protein assays may be envisioned for use in a BAV management protocol to allow for clinical risk monitoring of BAV patients. For instance, regular sampling of plasma levels of extracellular matrix proteins in previously diagnosed BAV may be used as an indication of the presence of vessel wall injury. In this population such a finding puts the immediate suspicion on injury localized in the aorta, and imaging tests may be indicated in patients with consistently elevated plasma levels to diagnose dilatation, and/or risk for accelerated dilatation if highly abnormal flow patterns are observed. Clinical risk stratification of BAV patients is much desired considering the variation in development and progression of aortic dilatation and the increased risk for serious complications including sudden cardiac death [18-20]. Detailed analysis of abnormal blood flow patterns and MMP 2 plasma levels of extracellular matrix proteins may enable earlier, better, and individually tailored intervention in high-risk groups with an anticipated reduction in morbidity and mortality within this important class of congenital malformations.

### Limitations of the Study

Due to the relatively small number of samples and lack of age stratification in our data the results do not yield insight into whether misdirected flow patterns are a cause or a consequence of aortic dilatation. This preliminary study aimed at investigating 3-D CMR flow analysis as a promising discriminating test for at-risk patients. Despite the small sample some important significant correlations were elucidated, but a larger study, spanning a more complete spectrum of ages and flow patterns, and aortic dimensions, will be required to investigate causality of these phenomena.

Another limitation of the study is the relatively simplistic and naïve quantification of abnormality the aortic flow jet by calculation of the flow angle. The flow jet angle, investigated in this work, quantifies mis-direction of aortic flow, presumably due at least in part by incomplete opening of the aortic valve. While this phenomenon is readily observed and quantified by velocity-encoded CMR as we have shown in this paper, it only quantifies an important but limited and simple aspect of the relevant fluid mechanics at work. More complete and sophisticated characterization of the hemodynamic stresses and forces acting on the aorta in this (and other) congenital disease are quite challenging from aspects of both mathematics and imaging technology.

Increasing availability of 4-D flow imaging methods on commercial platforms, along with emerging research thrusts integrating CMR flow data with computational flow modeling, are anticipated to further improve on the accuracy and precision of the ad-hoc methods employed in this early investigation. We believe that combination of computational fluid modeling and 4-D blood flow CMR, although currently still in an early stage of development, carries great potential to contribute important new knowledge and answers to many important questions involving blood flow in congenital heart disease.

## Conclusions

In conclusion, the results of this study suggest that abnormal flow patterns, abnormal protein expression and aortic dilatation are all directly related in BAV patients. Our findings support the hypothesis that both factors are acting simultaneously in the development of dilatation [49]. Despite small numbers of subjects significant correlation was established in our data between the systolic LV outflow jet angle with the aortic root channel and dilatation at the levels of the sinus of Valsalva, sinotubular junction, and the ascending aorta. Significant correlation was observed of plasma levels of MMP 2 with dilatation at the ascending aorta level, and with the flow jet angle. Although it remains inconclusive from this study whether abnormal aortic flow patterns and extracellular matrix protein expression are a cause or a consequence of aortic dilatation, this study illustrates the potential future use of plasma biochemical markers as non-specific screening parameters, and quantitative imaging metrics of abnormal 3-D blood flow imaging as possible localized predictors for aortic dilatation.

## Competing interests

The authors declare that they have no competing interests.

## Authors' contributions

PMdR performed much of the final data analysis, the MMP analyses, and drafted the manuscript; DSIII performed/supervised and read most CMR scans, was responsible for clinical direction and project design, and participated in scan and analysis protocol design, and contributed in manuscript writing and review; PvdV participated in design of image analysis protocols, performed early image and statistical analysis work; ERZ participated in scan protocol and project design, and early image analysis efforts; WJP performed/supervised/read CMR scans, and participated in scan protocol design; SR performed all off-line image reconstructions, image transfer and management, and 4-D flow acquisition; TQR contributed software/methods development for flow visualization and analysis and HIPAA compliance; GD performed all final image analysis work; CL was responsible for patient recruitment and IRB matters; RPB contributed as research advisor for PMdR, PvdV, and ERZ; MEB served as project director, finalized manuscript preparation, designed scan and image analysis software and methods, and performed the final statistical analyses.
